# Small Activating RNAs: Towards the Development of New Therapeutic Agents and Clinical Treatments

**DOI:** 10.3390/cells10030591

**Published:** 2021-03-08

**Authors:** Hossein Ghanbarian, Shahin Aghamiri, Mohamad Eftekhary, Nicole Wagner, Kay-Dietrich Wagner

**Affiliations:** 1 Cellular and Molecular Biology Research Center, Shahid Beheshti University of Medical Sciences, Tehran 19857-17443, Iran; hghanbarian@sbmu.ac.ir; 2 Department of Medical Biotechnology, School of Advanced Technologies in Medicine, Shahid Beheshti University of Medical Sciences, Tehran 19839-63113, Iran; eftekharymohamad@gmail.com; 3 Student Research Committee, Department of Medical Biotechnology, School of Advanced Technologies in Medicine, Shahid Beheshti University of Medical Sciences, Tehran 19839-63113, Iran; shahin.aghamiri@sbmu.ac.ir; 4 Université Côte d’Azur, CNRS, INSERM, iBV, 06107 Nice, France

**Keywords:** small interfering RNAs (siRNAs), microRNAs (miRNAs), small activating RNAs (saRNA), molecular mechanism, neuronal development, cardiac development, cancer, therapeutics

## Abstract

Small double-strand RNA (dsRNA) molecules can activate endogenous genes via an RNA-based promoter targeting mechanism. RNA activation (RNAa) is an evolutionarily conserved mechanism present in diverse eukaryotic organisms ranging from nematodes to humans. Small activating RNAs (saRNAs) involved in RNAa have been successfully used to activate gene expression in cultured cells, and thereby this emergent technique might allow us to develop various biotechnological applications, without the need to synthesize hazardous construct systems harboring exogenous DNA sequences. Accordingly, this thematic issue aims to provide insights into how RNAa cellular machinery can be harnessed to activate gene expression leading to a more effective clinical treatment of various diseases.

## 1. Introduction

Small interfering RNAs (siRNAs) and microRNAs (miRNAs), key regulators of gene expression, are recognized as small double-stranded RNA (dsRNA) molecules [1,2,3]. They are loaded onto Ago proteins generating an active Ago–RNA complex, which regulates gene expression at transcription and post-transcription levels [4,5]. Several studies have demonstrated that dsRNAs mostly inhibit gene expression either by chromatin modifications or by translation inhibition [6,7,8,9]. A new kind of small dsRNA, small activating RNAs (saRNAs), are able to induce rather than inhibit gene expression by targeting promoter sequences of some genes, which is termed “RNA activation” (RNAa) [10,11,12,13]. saRNAs are 21 nucleotides in length and act in an Ago2-dependent manner in mammals similar to RNAi [11]. Although the first reports showed that gene expression can be activated by synthetic dsRNAs targeting promoter regions, more recent studies confirmed that RNAa is an evolutionarily conserved mechanism present in diverse eukaryotic organisms ranging from nematodes to humans [14,15]. Like small double-stranded activating RNAs, it has been evidenced that some miRNAs activate gene expression through targeting both promoter sequence and/or AU-rich elements in UTRs [11,16,17]. Several independent studies have shown that a new group of activating RNAs could be generated from natural antisense transcripts (NATs) [14,15,16]. Antisense transcripts, known as long non-coding RNAs, are transcribed from the opposite DNA strand of a protein-coding gene locus, which is complementary to the corresponding coding RNA. They are functional elements, expressed in a tissue-specific manner, and generally low in abundance [17]. Recent reports demonstrate that antisense transcripts regulate their sense (protein-coding) partners through diverse transcriptional and post-transcriptional mechanisms [18,19]. While most antisense transcripts suppress corresponding sense gene expression, they can also be regarded as targets for saRNAs [13,15]. Notably, blocking the interaction of the sense (mRNA) and antisense transcripts (forming a natural duplex) and/or destruction of antisense transcripts by siRNAs or single-stranded oligonucleotides (antagoNATs) results in locus-specific transcriptional de-repression and upregulation of the gene, possibly by activation of the RNAi machinery [14,15,16]. Apart from promoter sequences, which are known as the main target sites for saRNAs, gene activation by small RNA fragments could operate in any genomic region where antisense transcripts are presented. Despite tremendous advantages offered by saRNAs, there are still critical challenges limiting the development of these cutting-edge therapeutics. The most significant challenges include low siRNA stability, degradation and opsonization in the bloodstream, and off-target effects (Table 1). Nanoparticle-mediated delivery systems can open a new way to overcome these problems.

In this review, we present recent advances and challenges in the therapeutic applications of saRNAs.

## 2. Small Activating RNAs Are Involved in Locus-Specific Induction of Neural Genes

saRNA-mediated locus-specific gene activation has been studied in neural cells. Based on many studies, two models of locus-specific gene activation have been reported, promoter-targeted duplex RNA activating and natural antisense disruption, leading to changes in chromatin structure [8,14,15,20]. Several studies show that endogenous saRNAs selectively activate gene expression in neurons through targeting promoter sequences [21,22]. Kuwabara et al. have reported a neural-specific dsRNA of about 20 bp containing the NRSE (neuron restrictive silencer element) sequence. This sequence, which is defined as the NRSE/RE1, is localized within promoter regions of neuron-specific genes and is recognized by neuronal restricted silencing factor/RE-1 silencing transcription factor (NRSF/REST) leading to neuron-specific gene suppression in a non-neuronal cell. During an early stage of neurogenesis, the NRSE dsRNA induces the expression of genes containing the NRSE/RE1 sequence in their promoters. Indeed, the noncoding dsRNA, as an endogenous activating RNA, interacts with NRSF/REST machinery and modulates its function. Through this process, neural stem cells can be differentiated into neuronal and glial cells [21]. Cell-mediated brain repair suffers from poor survival rate of transplanted cells and the low efficiency of differentiation into neuronal cells [23]. Diodato and his colleagues have used pre-miRNAs (as activating RNAs) to increase the expression of Emx2, a human homeobox transcription factor modulating a number of developmental mechanisms such as development of cerebral cortex. Their results showed that the transactivation of Emx2 can result in delayed differentiation, self-renewal, and decreased death of neuronally committed precursors [24]. Exogenous saRNA-mediated gene activation in the brain has been reported by Fimiani and his co-workers [22]. Synthesized saRNAs induce the Foxg1 transcription factor, a key regulator of cortico-cerebral development and function. Foxg1 allele duplication and deletion in humans results in West and Rett syndromes, respectively [25]. As a prospective RNAa therapy of Rett syndrome, Foxg1 gene expression in neural cells has been induced in vitro and in vivo by intraventricular injection of saRNA to mouse neonates [22]. NATs destruction is another alternative locus-specific gene activation mechanism, which holds great therapeutic promise. For example, Modarresi et al. have studied the regulatory role of a natural antisense transcript in the brain-derived neurotrophic factor (BDNF) locus [14]. BDNF is a member of the neurotrophin growth factor family and is essential for neuronal maturation, plasticity, memory processes, and differentiation [26,27,28,29,30,31]. Interestingly, locus-specific induction of Bdnf expression is evidenced, both in vitro and in vivo, following degradation of a natural antisense transcript by single-stranded oligonucleotides (term antagoNAT) or siRNAs [14]. Induction of endogenous Bdnf expression following Bdnf-AS repression in neurospheres induced neuronal progenitor cell differentiation. Corresponding to these in vitro experiments, in vivo Bdnf mRNA and protein expression is also induced upon intracerebroventricular injection of Bdnf-antagoNAT9 in mice. It seems that in the case of Bdnf induction, a RNAa mechanism applies through regulating epigenetic modifications, namely reduction of the H3K27me3 repressive mark at the Bdnf locus. Transient induction of neurotrophin expression using the RNAa system is suggested as a pharmacological approach for several neurological disorders, as reduced neurotrophin expression has been observed in different neurodegenerative and neurodevelopmental disorders [14].

Spinal muscular atrophy (SMA) is another example of a neuromuscular disorder [32]. Insufficient expression of functional survival motor neuron protein (SMN), which is correlated with disease severity, leads to muscle weakness after birth [33,34,35]. Several therapeutic efforts have focused on increasing SMN expression [32]. d’Ydewalle et al. have identified a natural antisense transcript in SMN (SMN-AS) locus, which transcriptionally suppresses SMN expression through epigenetic modifications. Importantly, knockdown of the antisense transcript induces SMN transcriptional activity either in patient-derived cells or in the central nervous system of a SMA mouse model in vivo, improving survival of the mice and indicating a novel therapeutic target for SMA [36].

We believe that the next step for clinical translation of RNAa therapeutics for the treatment of various neurodegenerative and neurodevelopmental disorders is the development of novel drug delivery systems.

## 3. Small Activating RNAs Are Involved in Locus-Specific Induction of Cardiac Genes

RNAa-mediated locus-specific activation of gene transcription has been reported in cardiovascular cells [37,38,39]. In all reported studies, targeting of promoter regions by small dsRNA or small hairpin RNA (shRNA) leads to transcriptional activation of cardiovascular genes, which could open the way for therapeutic strategies. For instance, targeting the promoter region of vascular endothelial growth factor (VEGF) by shRNA results in transcriptional activation, suggesting a therapeutic strategy for myocardial infarction [37]. Targeting the antisense transcript has been recently demonstrated as an alternative RNA activating system in the cyclin-dependent kinase 9 (Cdk9) locus, a key player in cardiac development [15,16]. Cdk9 is associated with specific cyclins to form a heterodimer, Cyclin T/Cdk9, which is also known as the positive transcription elongation factor-b (P-TEFb) [40,41,42]. P-TEFb activates the polymerase II transcription machinery via phosphorylation of the carboxyl-terminal domain (CTD) [43,44]. Therefore, Cdk9 is mainly involved in transcriptional regulation and plays a critical role in several differentiation pathways. Furthermore, Cdk9 regulates cardiac-specific genes including Nkx2.5, Anf, and ß-Myh via interactions with the p300/GATA4 complex, particularly involved in cardiac differentiation [45]. Moreover, we have shown recently that Cdk9 regulates apoptosis in cardiomyocytes by modulating miRNA-1 expression, a critical microRNA for cardiac differentiation [46,47,48]. It is therefore possible that both synthesis and activity of Cdk9 are tightly regulated at the transcriptional and post-transcriptional levels. In this regard, at least three non-coding RNAs are involved in Cdk9 regulation [16]. In the context of normal human cardiomyocytes, Cdk9 activity is suppressed at the protein level via interaction with 7SK non-coding RNA and at the translational level through muscle-specific microRNAs, specifically miR-1 and miR-133 [46,49,50]. We have recently reported a third mode of RNA control in the Cdk9 locus [15]. Small non-coding RNA molecules of 22bp with sequences homologous to the transcript result in transcriptional activation of Cdk9. Interestingly, NATs complementary to the most 3′ and 5′ regions of the gene were identified. Indeed, hybridization of the short single-stranded cognate transcript fragments with antisense transcripts provides the signal for transcriptional activation. The requirement of Argonaute proteins and endogenous antisense transcripts for transcriptional activation indicates that the activating single-stranded small RNAs are processed by the RNAi machinery [51,52]. Similar to siRNA knockdown, antisense transcript distraction following the sense oligoribonucleotide electroporation could represent a secondary phenomenon of the activation of the RNAi machinery. This activation may then result in a change in epigenetic modifications at the locus, leading to the induction of Cdk9 transcription as described for several genes [1,53]. As a functional consequence of RNA activation in the Cdk9 locus, an increased cardiac differentiation potential is observed in ES cells when electroporated with the sense oligoribonucleotide. Interestingly, injection into wild-type blastocysts of RNA-programmed ES cells contributes specifically to heart development in vivo, indicating that a transient RNA activation system is sufficient to create a cardiac differentiation “memory” in cells and may represent a novel tool for RNA–cell reprogramming applied in regenerative medicine.

## 4. Small Activating RNAs: New Insights into Cancer Therapy

Recently, RNA-based therapeutics have gained more attention in cancer therapy due to their enormous potential to selectively target previously undruggable genes and gene expression modulators. Unlike RNAi, which mostly targets sense transcripts, RNAa, as an alternative and promising new therapeutic strategy, can activate gene expression in a natural manner by targeting antisense transcripts or promoter regions [13,54,55,56]. Tumor suppressor genes, which are mostly suppressed in cancers, could be targeted by saRNA to enhance transcriptional activation and restore a normal cell phenotype.

**Figure 1:**
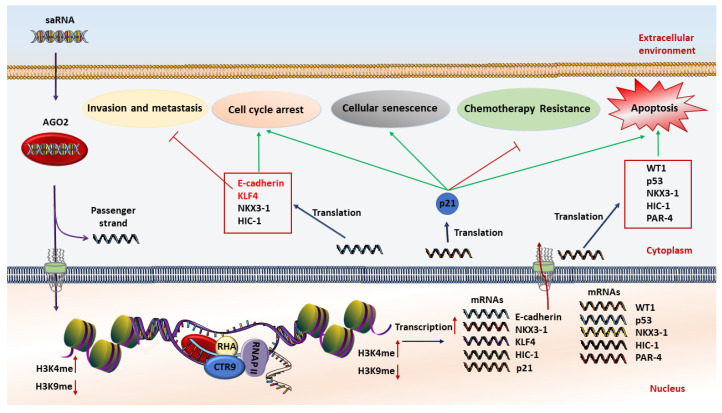
Schematic illustration of the anticancer mechanism of saRNA (small activating RNA)-based therapeutics. At first, saRNAs are loaded on the AGO2 protein. Then AGO2 separates the passenger strand. After that, the complex of saRNA guide strand and AGO2 cross the nuclear and interact with promoter sequences of interested genes to increase transcription by methylation of H3K4 and/or demethylation of H3K9. The expression level of tumor suppressor genes is restored, resulting in induction of apoptosis, chemoresistance reversal, inhibition of invasion and metastasis, cell cycle arrest, and cellular senescence.

saRNAs can exert their anticancer effects through induction of cell cycle arrest, cellular senescence, proliferation inhibition, apoptosis induction, metastasis suppression, and multidrug resistance reversal (Figure 1). Although several tumor suppressor genes including E-cadherin, NKX3-1, Wt1, and P53 have been induced by this method, p21 is the most investigated tumor suppressor gene for RNAa-mediated gene activation in several tumors and cell lines [10,57,58,59,60,61,62]. P21 is a negative regulator of the cell cycle and is rarely mutated in cancers, and therefore represents a key target for small RNA activation cancer therapy. In this regard, re-activation of the p21 gene by targeting promoter regions inhibits cell viability and proliferation rates, while it induces apoptotic cell death and sensitizes lung cancer cells to chemotherapeutic agents, providing a new approach in cancer therapy [59,60,63].

## 5. Towards the Development of New Therapeutic Agents

In the past few years, a steadily increasing number of clinical and preclinical studies have been performed, using various saRNA-based therapies for the treatment of a multitude of different diseases (Table 2 and Supplementary Table S1).

Due to their potential involvement in human disorders, strong efforts have been undertaken to develop new therapeutic agents applying a RNAa strategy. For instance, two companies in the USA, RNAa Therapeutics and OPKO-CURNA, are investigating therapeutic approaches based on RNA activation. Of note, mipomersen, an oligonucleotide targeting apolipoprotein B, was approved by the FDA in January 2013, but received a negative opinion from a European Medicines Agency panel [90]. RNA-activating molecules as promising drugs are under active investigation due to their high potency and specificity, locus-specific manner functions, targeting of the correct cells, small molecular size, and low toxicity [10,91]. Traditionally, an exogenous DNA construct is often required for ectopic gene expression. Apart from exogenous DNA constructs excluding regulatory elements, such systems have been problematic in the clinic because of requiring viral-based vectors for gene delivery. Several limitations and undesirable side effects of viral-based gene therapy including host genome integrity, the tedious process of construction, and various negative immunological responses which have been previously reported [92,93,94,95]. Upon delivery as mature moieties, an RNAa system offers a more natural approach and safer gene therapy method by targeting promoter regions or through natural antisense destruction, which activates endogenous targeted gene expression in a locus-specific manner in the absence of exogenous DNA. Moreover, RNAa offers an endogenous induction in a natural cellular niche leading to the correctly processed proteins with their appropriate modifications. Notably, in the case of gene activation through natural antisense destruction, short oligoribonucleotides derived from a sense transcript (mRNA) can function as an activator RNA [15], which are safer, natural, and without activating immune responses.

To improve the medicinal properties and potential in vivo applications of saRNAs, two steps must be taken. First, novel nanoparticle-based drug delivery systems must be developed to increase the drug accumulation in the targeted tissue and also address the aforementioned challenges in the therapeutic application of saRNAs. To date, different delivery systems have been developed for the delivery of saRNAs. Recently, lipid-based nanoparticles (LNPs) have attracted worldwide attention. LNP-based delivery systems face serious limitations such as toxicity, low thermodynamic stability, poor efficiency of encapsulation, and leaking challenges [96]. To overcome these limitations, various strategies have been proposed. The surface modification of delivery systems with flexible, non-ionic, and hydrophilic polymers, such as PEG, has been proposed as a robust strategy to address serum protein opsonization issues [97,98]. Furthermore, surface modification with biodegradable nano-polymers such as PEG can be used to decrease the toxicity of nanoparticles [99]. To increase the expression of P21, 2′-fluoro-modified P21 saRNA (dsP21-322-2′F) was delivered into an orthotopic bladder cancer mouse model by using a novel PEG-modified lipid nanoparticle. Results showed a significant increase in urothelium uptake and high tumor shrinkage [80]. Aptamers, nucleic acid ligands, can be used for targeted drug delivery as they can form specific three-dimensional structures based on their sequences. Yoon and his colleagues have synthesized PDAC specific 2′-Fluropyrimidine RNA-aptamers (2′F-RNA)- P19 and P1 for targeted delivery of saRNA into both PANC-1 and AsPC-1 engrafted mice. After intravenous injections of the aptamer–C/EBPα saRNA in both tumor mice models, tumor growth was significantly suppressed in comparison with mice treated with gemcitabine [84].

The second step for the development of effective saRNA therapeutics relies on tailoring site-specific chemical modifications of saRNAs. For instance, blocking the 5′-OH of the passenger strand and modifications to the 2′ backbone (i.e., 2′-OMe, 2′-Fluoro, and locked nucleic acid) has been demonstrated to reduce its off-target potential and increase endonuclease resistance and serum stability, respectively [55,58,100]. With single-stranded therapeutic oligonucleotides, designated antagoNAT, chemical modifications not only promote metabolic stability and target specificity but also minimize the length of the oligonucleotide to improve cellular uptake. In this case, 16-mer antagoNAT oligonucleotides, also known as a gapmer [101], with three locked nucleic acid (LNA) substitutions at each end and phosphorothioate-modified backbones, have been used in in vivo studies [14,102,103,104,105]. Along with chemical modifications to improve the stability and specificity, targeted and efficient in vivo delivery of oligonucleotides is also critical for RNA-based therapies. Among the currently investigated approaches [106,107,108,109], lipid-based formulations are the most promising delivery agents for systemic or localized saRNA delivery [100,110,111]. Several clinical trials that are testing RNAa-based drugs are commonly using lipid carriers [112,113]. Considering the increasing knowledge regarding RNAa-based locus-specific gene activation as a new strategy with promising therapeutic perspectives, it is clear that there is a growing scientific as well as a commercial interest to develop new therapeutic agents and clinical treatments based on this innovative approach. Lipid nanoparticle-formulated nucleoside-modified RNAs have been recently introduced as the Covid-19 vaccine, supporting the notion of efficient lipid-based delivery, safety, and efficiency of RNA therapies [114,115].

## 6. Conclusions and Future Perspectives

RNAa-based drugs have gained great attention in the past few years owing to their high potential in treating various disorders. However, delivery challenges slow down the clinical translation of this new class of therapeutic agents. Enzymatic digestion, low cellular uptake, poor endosomal escape, off-target effects, and fast clearance from the bloodstream have necessitated optimization of not only the RNAa molecules but also of the delivery systems. Many non-viral delivery systems have been designed and developed for the delivery of RNAa-based therapeutic systems. Nanoparticle-based delivery systems have several advantages including high functionalization and targeting capacity and ease of large-scale development, which make them a promising delivery system for medical applications. For instance, several studies demonstrated that PEGylation of different materials such as PEI can enhance their circulation time and reduce hemolysis and serum opsonization. Chemical modifications of RNA molecules herald a new era in the development of these cutting-edge RNA-based therapeutics. However, these modifications might be limited due to the occurrence of severe side effects and functionality reduction, which are topics that urgently need to be addressed.

Future applications of RNAa-based therapeutics will significantly advance due to the progress in the development of drug delivery vehicles, reduction of the off-targeting problem, and huge large-scale synthesis capacity that will be offered by pharmaceutical companies. The combinatorial strategy using RNAa with chemotherapeutic drugs also could be a new strong treatment modality for different types of diseases.

## Figures and Tables

**Table 1:** Pros and cons of using saRNAs as therapeutics.

Advantages	Disadvantages
Effective gene activation	Poor cellular uptake
Locus-specific activation of gene transcription, including undruggable targets	High sensitivity to RNase degradation
Easy to manufacture	Renal clearance
Cost-effectiveness	Repeated administration
Low toxicity	Off-target effects
Easy large-scale production	Activation of Toll-like receptors
Poor immunogenicity	

**Table 2:** Significant preclinical and clinical studies on the features of saRNA-based therapeutics.

Disease Condition	Gene	Comments	Ref.
Advanced liver cancer	CEBPA	The first clinical trial for saRNA-based therapeutics (NCT ID: NCT02716012; company: Mina Alpha Limited; phase 1). MTL-CEBPA shows favorable safety and promising synergistic effects in combination with TKIs.	[64]
Adult solid tumors	CEBPA	A new clinical trial of MTL-CEBPA in combination with pembrolizumab (NCT ID: NCT04105335; Phase 1; recruitment status: Recruiting).	[65]
Prostate cancer	P21	Proliferation inhibition and tumor shrinkage.	[58]
Hepatocellular carcinoma (HCC)	P21	Cell cycle arrest and inhibition of invasion and migration.	[10]
Non-small-cell lung carcinomas	P21	In vitro: Proliferation inhibition, cell cycle arrest, and apoptosis induction. In vivo: Inhibition of tumor growth.	[59]
Pancreatic cancer	P21	In vitro: Proliferation inhibition, cell cycle arrest, and apoptosis induction. In vivo: Inhibition of tumor growth; high safety.	[66]
Bladder cancer	P21	Proliferation inhibition, cell cycle arrest, and apoptosis induction.	[67]
HCC	WT1	Proliferation inhibition and apoptosis induction.	[68]
Prostate cancer	Ecad	Inhibition of invasion and migration.	[69]
Bladder cancer	Ecad	Inhibition of invasion and migration.	[70]
Breast cancer	Ecad	In vitro: Proliferation inhibition, cell cycle arrest, apoptosis induction, and inhibition of invasion and migration. In vivo: Tumor growth inhibition	[71]
Prostate cancer	KLF4	Proliferation inhibition, cell cycle arrest, apoptosis induction, and inhibition of invasion and migration.	[72]
Malignant pheochromocytoma	TP53	In vitro: Cell cycle arrest, proliferation inhibition, and apoptosis induction. In vivo: Tumor shrinkage.	[73]
Breast cancer	HIC-1	Proliferation inhibition and apoptosis induction.	[74]
Bladder and prostate cancer	PAWR	Proliferation inhibition and apoptosis induction.	[75]
Prostate cancer	NKX3-1	In vitro: Proliferation inhibition, cell cycle arrest, apoptosis induction. In vivo: Tumor growth inhibition.	[76]
Nephrolithiasis	TRPV5	In vitro: TRPV5 expression induction. In vivo: TRPV5 expression induction and reduction in the formation of CaOx kidney stone.	[77]
Renal cell carcinoma	VHL	Cell growth inhibition and apoptosis induction.	[78]
HCC	NIS	Apoptosis induction and viability reduction of cancer cells.	[79]
Bladder cancer	P21	Tumor Shrinkage	[80]
HCC	CEBPA	In vitro: CEBPA overexpression. In vivo: Tumor growth inhibition and tumor shrinkage.	[64]
HCC	CEBPA	In vitro: Proliferation inhibition. In vivo: Tumor burden reduction.	[81]
HCC	CEBPA	In vitro: Cell migration and invasion inhibition. In vivo: Metastasis inhibition	[82]
Colorectal cancer	P21	In vitro: Apoptosis induction, proliferation inhibition, and cell migration and invasion inhibition. In vivo: Tumor growth inhibition.	[83]
Pancreatic ductal adenocarcinoma	CEBPA	In vitro: Proliferation inhibition. In vivo: Tumor shrinkage.	[84]
Prostate cancer	DPYSL3	In vitro: Proliferation inhibition and cell migration and invasion inhibition. In vivo: Metastasis inhibition	[85]
Diabetes-induced erectile dysfunction	Nos2	In vitro: iNos overexpression. In vivo: iNos overexpression and enhancement of peak intracavernous pressure.	[86]
Human metastatic castration-resistant prostate cancer	Notch1	In vitro: Cell migration and invasion suppression, cell cycle arrest, and apoptosis inhibition. In vivo: Tumor growth inhibition and suppression of VEGF and AR pathways mechanisms.	[87]
Non-alcoholic fatty liver disease	HNF4A	In vitro: Increase in the expression level of HNF4A, CYP450, CYP3A4, CYP3A5, and CYP3A7. In vivo: Liver triglyceride reduction, high-density lipoprotein/low-density lipoprotein (HDL/LDL) ratio enhancement, and white adipose tissue/body weight ratio reduction.	[88]
Endometrial carcinoma	FHIT	Proliferation, invasion, and metastasis inhibition.	[89]

Supplementary Table S1 provides more experimental details on the same studies mentioned in Table 2.

## Supplementary Materials

The following are available online at https://www.mdpi.com/2073-4409/10/3/591/s1, Table S1: Significant preclinical and clinical studies on the features of saRNA-based therapeutics—Extended characteristics.
