# Supplementary material for: Small Activating RNAs: Towards the Development of New Therapeutic Agents and Clinical Treatments
Source: Cells. 2021 Mar 8;10(3):591. doi: 10.3390/cells10030591 (PMC8001863; doi:10.3390/cells10030591)
Supplement: Supplementary file 1 [file cells-10-00591-s001.pdf]

Table S1. Significant preclinical and clinical studies on the features of saRNA-based therapeutics - extended characteristics.

saRNA	Disease Condition	Delivery system	Gene	*In vitro*	*In vivo*	Cell lines	Route of administration	Animal models	Comments	Ref.
MTL-CEBPA	Advanced liver cancer	SMARTICLES (liposome)	CEBPA	-	-	-	Intravenous	-	The first clinical trial for saRNA-based therapeutics (NCT ID: NCT02716012; company: Mina Alpha Limited; phase 1). MTL-CEBPA shows favorable safety and promising synergistic effects in combination with TKIs.	[1]
MTL-CEBPA in combination with pembrolizumab	Adult solid tumors	SMARTICLES	CEBPA	-	-	-	Intravenous	-	A new clinical trial of MTL-CEBPA in combination with pembrolizumab (NCT ID: NCT04105335; Phase 1; recruitment status: Recruiting)	[2]
dsP21-322	Prostate cancer	Lipidoid-encapsulated nanoparticle (LNP)	P21	+	+	PC3	Intratumoral	PC3-bearing tumor-xenograft nude mice model	Proliferation inhibition and tumor shrinkage.	[3]
dsP21-322	Hepatocellular carcinoma (HCC)	Lipofectamine RNAiMax reagent (Life Technologies)	P21	+	-	HepG2 and Hep3B	-	-	Cell cycle arrest and inhibition of invasion and migration.	[4]
dsP21-322	Non-small-cell lung carcinomas	LipofectamineTM2000	P21	+	+	A549	Intratumoral	A549 subcutaneously transplanted BALB/c nude mice	*In vitro*: Proliferation inhibition, cell cycle arrest, and apoptosis induction.<br>*In vivo*: Inhibition of tumor growth.	[5]
dsP21-322	Pancreatic cancer	Lipofectamine 2000	P21	+	+	PANC-1	Intratumoral	PANC-1 subcutaneously transplanted nude mice	*In vitro*: Proliferation inhibition, cell cycle arrest, and apoptosis induction.<br>*In vivo*: Inhibition of tumor growth; high safety.	[6]
dsP21-322	Bladder cancer	Lipofectamine 2000	P21	+	-	T24	-	-	Proliferation inhibition, cell cycle arrest, and apoptosis induction.	[7]

dsWT1-319	HCC	Lipofecta mine 2000	WT1	+	-	HepG2	-	-	Proliferation inhibition and apoptosis induction.	[8]
dsEcad-215	Prostate cancer	Lipofecta mine 2000	E-cadheri n	+	-	PC3	-	-	Inhibition of invasion and migration.	[9]
dsEcad-215	Bladder cancer	Lipofecta mine 2000	E-cadheri n	+	-	5637	-	-	Inhibition of invasion and migration.	[10]
dsEcad-215, 302	Breast cancer	Lipofecta mine 2000	E-cadheri n	+	+	MDA-MB-453 and MCF-7	Intratumoral	MDA-MB-453 subcutaneously injected into the BALB/c nude mice	*In vitro*: Proliferation inhibition, cell cycle arrest, apoptosis induction, and inhibition of invasion and migration.<br>*In vivo*: Tumor growth inhibition	[11]
dsKLF4-496	Prostate cancer	Lipofecta mine 2000	KLF4	+	-	PC-3 and DU145	-	-	Proliferation inhibition, cell cycle arrest, apoptosis induction, and inhibition of invasion and migration.	[12]
dsP53-285	Malignant pheochrom ocytoma	Lipofecta mine RNAiMax	TP53	+	+	PC12	PC12 cells were infected with Lenti-dsP53-285 and subcutaneously injected	PC12 subcutaneously injected xenograft mice model	*In vitro*: Cell cycle arrest, proliferation inhibition, and apoptosis induction.<br>*In vivo*: Tumor shrinkage.	[13]
dsHIC1-2998	Breast cancer	Lipofecta mine 2000	HIC-1	+	-	MCF-7 and MDA-MB-231	-	-	Proliferation inhibition and apoptosis induction	[14]
dsPAWR-435	Bladder cancer and prostate cancer	Lipofecta mine 2000	PAWR	+	-	T24 and PC3	-	-	Proliferation inhibition and apoptosis induction	[15]
dsNKX3-1-381	Prostate cancer	Lipofecta mine 2000	NKX3-1	+	+	PC-3, CWR22R, LAPC4, and CWR22R V1	Intratumoral	PC3-bearing tumor-xenograft mice model	*In vitro*: Proliferation inhibition, cell cycle arrest, apoptosis induction.<br>*In vivo*: Tumor growth inhibition.	[16]
saRNA ds-320	Nephrolithi asis	Lipofecta mine 2000	TRPV5	+	+	NRK-52E	Retrograde ureteral injection	Calcium oxalate stone-forming rat model	*In vitro*: TRPV5 expression induction.<br>*In vivo*: TRPV5 expression induction and reduction in the formation of CaOx kidney stone.	[17]

dsVHL-821	Renal cell carcinoma	Lipofectamine RNAiMax	VHL	+	-	769-P	-	-	Cell growth inhibition and apoptosis induction.	[18]
saRNA-482	HCC	Lipofectamine RNAiMax	NIS	+	-	HepG2 and Hep3B	-	-	Apoptosis induction and viability reduction of cancer cells.	[19]
dsP21-322-2'F	Bladder cancer	lipid nanoparticles	P21	-	+	-	Intravesical	Orthoptic bladder cancer mouse	Tumor Shrinkage	[20]
MTL-CEBPA	HCC	SMARTICLES	CEBPA	+	+	AML12, Clone 9, and HEPG2	Intravenous	Diethylnitrosamine-induced cirrhotic HCC rat, carbon-tetrachloride induced liver injury, and fibrosis mouse, and Methionine and choline deficient diet induced nonalcoholic steatohepatitis mouse	*In vitro*: CEBPA overexpression.<br>*In vivo*: Tumor growth inhibition and tumor shrinkage.	[21]
C/EBPa-saRNA	HCC	Poly(amidoamine) (PAMAM) dendrimers	CEBPA	+	+	HepG2	Intravenous	Rat liver tumor model	*In vitro*: Proliferation inhibition.<br>*In vivo*: Tumor burden reduction.	[22]
C/EBPa-saRNA	HCC	PAMAM dendrimers	CEBPA	+	+	HepG2 and SMMC-7721	Intravenous	liver orthotopic xenograft nude mice tumor model	*In vitro*: Cell migration and invasion inhibition.<br>*In vivo*: Metastasis inhibition	[23]
dsP21-322	Colorectal cancer	Lipopolyplex (HA anchored PE tumor-selective liposome)	P21	+	+	HT-29	Rectal	Orthotopic-HT29-bearing tumor-xenograft g NOD-SCID mice model colorectal tumor model	*In vitro*: Apoptosis induction, proliferation inhibition, and cell migration and invasion inhibition.<br>*In vivo*: Tumor growth inhibition.	[24]

C/EBPa-saRNA	Pancreatic ductal adenocarcinoma	Aptamer	CEBPA	+	+	PANC-1	Intravenous	AsPC-1-bearing mouse xenograft model and PANC-1-bearing mouse xenograft model	*In vitro*: Proliferation inhibition.<br>*In vivo*: Tumor shrinkage.	[25]
saV2-9 RNA	Prostate cancer	Aptamer	DPYSL3	+	+	PC-3	Intraperitoneal	Orthotopic mouse model with xenograft prostate tumor cells	*In vitro*: Proliferation inhibition and cell migration and invasion inhibition.<br>*In vivo*: Metastasis inhibition	[26]
iNOS saRNA	Diabetes-induced Erectile dysfunction	Adenovirus	Nos2	+	+	CSMCs	Intracavernous	Streptozotocin induced diabetes rat model	*In vitro*: iNos overexpression.<br>*In vivo*: iNos overexpression and enhancement of peak intracavernous pressure.	[27]
Notch1-saRNA-1480	Human metastatic castration-resistant prostate cancer	Lipofectamine 3000	Notch1	+	+	PC-3	Intratumoral	PC3-bearing tumor-xenograft mice model	*In vitro*: Cell migration and invasion suppression, cell cycle arrest, and apoptosis inhibition.<br>*In vivo*: Tumor growth inhibition and suppression of VEGF and AR pathways mechanisms.	[28]
HNF4A-saRNA	Non-alcoholic fatty liver disease	PAMAM dendrimers	HNF4A	+	+	HepG2, Hep3B, and PLCPRF5	Intravenous	NAFLD rat Model (High fat diet rat)	*In vitro*: Increase in the expression level of HNF4A, CYP450, CYP3A4, CYP3A5, and CYP3A7.<br>*In vivo*: Liver triglyceride reduction, high-density lipoprotein/low-density lipoprotein (HDL/LDL) ratio enhancement, and white adipose tissue/body weight ratio reduction	[29]
FHIT-saRNA expression vector	Endometrial carcinoma	Lipofectamine 3000	FHIT	+	-	ISK, RL-952, HEC-1-B, and AN3 CA	-	-	Proliferation, invasion, and metastasis inhibition	[30]

References

1. Sarker, D.; Plummer, R.; Meyer, T.; Sodergren, M.H.; Basu, B.; Chee, C.E.; Huang, K.W.; Palmer, D.H.; Ma, Y.T.; Evans, T.R.J., et al. MTL-CEBPA, a Small Activating RNA Therapeutic Upregulating C/EBP-α, in Patients with Advanced Liver Cancer: A First-in-Human, Multicenter, Open-Label, Phase I Trial. *Clin Cancer Res* **2020**, *26*, 3936-3946, doi:10.1158/1078-0432.CCR-20-0414.
2. Kramer, E.D.; Abrams, S.I. Granulocytic Myeloid-Derived Suppressor Cells as Negative Regulators of Anticancer Immunity. *Front Immunol* **2020**, *11*, 1963, doi:10.3389/fimmu.2020.01963.
3. Place, R.F.; Wang, J.; Noonan, E.J.; Meyers, R.; Manoharan, M.; Charisse, K.; Duncan, R.; Huang, V.; Wang, X.; Li, L.C. Formulation of Small Activating RNA Into Lipidoid Nanoparticles Inhibits Xenograft Prostate Tumor Growth by Inducing p21 Expression. *Mol Ther Nucleic Acids* **2012**, *1*, e15, doi:10.1038/mtna.2012.5.

4. Kosaka, M.; Kang, M.R.; Yang, G.; Li, L.C. Targeted p21WAF1/CIP1 activation by RNAa inhibits hepatocellular carcinoma cells. *Nucleic Acid Ther* **2012**, *22*, 335-343, doi:10.1089/nat.2012.0354.
5. Wei, J.; Zhao, J.; Long, M.; Han, Y.; Wang, X.; Lin, F.; Ren, J.; He, T.; Zhang, H. p21WAF1/CIP1 gene transcriptional activation exerts cell growth inhibition and enhances chemosensitivity to cisplatin in lung carcinoma cell. *BMC Cancer* **2010**, *10*, 632, doi:10.1186/1471-2407-10-632.
6. Zhang, Z.; Wang, Z.; Liu, X.; Wang, J.; Li, F.; Li, C.; Shan, B. Up-regulation of p21WAF1/CIP1 by small activating RNA inhibits the in vitro and in vivo growth of pancreatic cancer cells. *Tumori* **2012**, *98*, 804-811, doi:10.1700/1217.13507.
7. Yang, K.; Zheng, X.Y.; Qin, J.; Wang, Y.B.; Bai, Y.; Mao, Q.Q.; Wan, Q.; Wu, Z.M.; Xie, L.P. Up-regulation of p21WAF1/Cip1 by saRNA induces G1-phase arrest and apoptosis in T24 human bladder cancer cells. *Cancer Lett* **2008**, *265*, 206-214, doi:10.1016/j.canlet.2008.02.014.
8. Qin, Q.; Lin, Y.W.; Zheng, X.Y.; Chen, H.; Mao, Q.Q.; Yang, K.; Huang, S.J.; Zhao, Z.Y. RNAa-mediated overexpression of WT1 induces apoptosis in HepG2 cells. *World J Surg Oncol* **2012**, *10*, 11, doi:10.1186/1477-7819-10-11.
9. Mao, Q.; Zheng, X.; Yang, K.; Qin, J.; Bai, Y.; Jia, X.; Li, Y.; Xie, L. Suppression of migration and invasion of PC3 prostate cancer cell line via activating E-cadherin expression by small activating RNA. *Cancer Invest* **2010**, *28*, 1013-1018, doi:10.3109/07357900802620844.
10. Mao, Q.; Li, Y.; Zheng, X.; Yang, K.; Shen, H.; Qin, J.; Bai, Y.; Kong, D.; Jia, X.; Xie, L. Up-regulation of E-cadherin by small activating RNA inhibits cell invasion and migration in 5637 human bladder cancer cells. *Biochem Biophys Res Commun* **2008**, *375*, 566-570, doi:10.1016/j.bbrc.2008.08.059.
11. Junxia, W.; Ping, G.; Yuan, H.; Lijun, Z.; Jihong, R.; Fang, L.; Min, L.; Xi, W.; Ting, H.; Ke, D., et al. Double strand RNA-guided endogeneous E-cadherin up-regulation induces the apoptosis and inhibits proliferation of breast carcinoma cells in vitro and in vivo. *Cancer Sci* **2010**, *101*, 1790-1796, doi:10.1111/j.1349-7006.2010.01594.x.
12. Wang, J.; Place, R.F.; Huang, V.; Wang, X.; Noonan, E.J.; Magyar, C.E.; Huang, J.; Li, L.C. Prognostic value and function of KLF4 in prostate cancer: RNAa and vector-mediated overexpression identify KLF4 as an inhibitor of tumor cell growth and migration. *Cancer Res* **2010**, *70*, 10182-10191, doi:10.1158/0008-5472.CAN-10-2414.
13. Lin, D.; Meng, L.; Xu, F.; Lian, J.; Xu, Y.; Xie, X.; Wang, X.; He, H.; Wang, C.; Zhu, Y. Enhanced wild-type p53 expression by small activating RNA dsP53-285 induces cell cycle arrest and apoptosis in pheochromocytoma cell line PC12. *Oncol Rep* **2017**, *38*, 3160-3166, doi:10.3892/or.2017.5993.
14. Zhao, F.; Pan, S.; Gu, Y.; Guo, S.; Dai, Q.; Yu, Y.; Zhang, W. Small activating RNA restores the activity of the tumor suppressor HIC-1 on breast cancer. *PLoS One* **2014**, *9*, e86486, doi:10.1371/journal.pone.0086486.
15. Yang, K.; Shen, J.; Xie, Y.Q.; Lin, Y.W.; Qin, J.; Mao, Q.Q.; Zheng, X.Y.; Xie, L.P. Promoter-targeted double-stranded small RNAs activate PAWR gene expression in human cancer cells. *Int J Biochem Cell Biol* **2013**, *45*, 1338-1346, doi:10.1016/j.biocel.2013.03.022.
16. Ren, S.; Kang, M.R.; Wang, J.; Huang, V.; Place, R.F.; Sun, Y.; Li, L.C. Targeted induction of endogenous NKX3-1 by small activating RNA inhibits prostate tumor growth. *Prostate* **2013**, *73*, 1591-1601, doi:10.1002/pros.22709.
17. Zeng, T.; Duan, X.; Zhu, W.; Liu, Y.; Wu, W.; Zeng, G. SaRNA-mediated activation of TRPV5 reduces renal calcium oxalate deposition in rat via decreasing urinary calcium excretion. *Urolithiasis* **2018**, *46*, 271-278, doi:10.1007/s00240-017-1004-z.
18. Kang, M.R.; Park, K.H.; Lee, C.W.; Lee, M.Y.; Han, S.B.; Li, L.C.; Kang, J.S. Small activating RNA induced expression of VHL gene in renal cell carcinoma. *Int J Biochem Cell Biol* **2018**, *97*, 36-42, doi:10.1016/j.biocel.2018.02.002.
19. Xia, W.; Li, D.; Wang, G.; Ni, J.; Zhuang, J.; Ha, M.; Wang, J.; Ye, Y. Small activating RNA upregulates NIS expression: promising potential for hepatocellular carcinoma endoradiotherapy. *Cancer Gene Therapy* **2016**, *23*, 333-340, doi:10.1038/cgt.2016.36.
20. Kang, M.R.; Yang, G.; Place, R.F.; Charisse, K.; Epstein-Barash, H.; Manoharan, M.; Li, L.C. Intravesical delivery of small activating RNA formulated into lipid nanoparticles inhibits orthotopic bladder tumor growth. *Cancer Res* **2012**, *72*, 5069-5079, doi:10.1158/0008-5472.Can-12-1871.
21. Reebye, V.; Huang, K.W.; Lin, V.; Jarvis, S.; Cutilas, P.; Dorman, S.; Ciriello, S.; Andrikakou, P.; Voutila, J.; Saetrom, P., et al. Gene activation of CEBPA using saRNA: preclinical studies of the first in human saRNA drug candidate for liver cancer. *Oncogene* **2018**, *37*, 3216-3228, doi:10.1038/s41388-018-0126-2.
22. Reebye, V.; Sætrom, P.; Mintz, P.J.; Huang, K.W.; Swiderski, P.; Peng, L.; Liu, C.; Liu, X.; Lindkaer-Jensen, S.; Zacharoulis, D., et al. Novel RNA oligonucleotide improves liver function and inhibits liver carcinogenesis in vivo. *Hepatology* **2014**, *59*, 216-227, doi:10.1002/hep.26669.

23. Huan, H.; Wen, X.; Chen, X.; Wu, L.; Liu, W.; Habib, N.A.; Bie, P.; Xia, F. C/EBPα Short-Activating RNA Suppresses Metastasis of Hepatocellular Carcinoma through Inhibiting EGFR/β-Catenin Signaling Mediated EMT. *PLoS One* **2016**, *11*, e0153117, doi:10.1371/journal.pone.0153117.
24. Wang, L.L.; Feng, C.L.; Zheng, W.S.; Huang, S.; Zhang, W.X.; Wu, H.N.; Zhan, Y.; Han, Y.X.; Wu, S.; Jiang, J.D. Tumor-selective lipopolyplex encapsulated small active RNA hampers colorectal cancer growth in vitro and in orthotopic murine. *Biomaterials* **2017**, *141*, 13-28, doi:10.1016/j.biomaterials.2017.06.029.
25. Yoon, S.; Huang, K.W.; Reebye, V.; Mintz, P.; Tien, Y.W.; Lai, H.S.; Sætrom, P.; Reccia, I.; Swiderski, P.; Armstrong, B., et al. Targeted Delivery of C/EBPα -saRNA by Pancreatic Ductal Adenocarcinoma-specific RNA Aptamers Inhibits Tumor Growth In Vivo. *Mol Ther* **2016**, *24*, 1106-1116, doi:10.1038/mt.2016.60.
26. Li, C.; Jiang, W.; Hu, Q.; Li, L.-C.; Dong, L.; Chen, R.; Zhang, Y.; Tang, Y.; Thrasher, J.B.; Liu, C.-B., et al. Enhancing DPYSL3 gene expression via a promoter-targeted small activating RNA approach suppresses cancer cell motility and metastasis. *Oncotarget* **2016**, *7*, 22893-22910, doi:10.18632/oncotarget.8290.
27. Wang, T.; Li, M.; Yuan, H.; Zhan, Y.; Xu, H.; Wang, S.; Yang, W.; Liu, J.; Ye, Z.; Li, L.C. saRNA guided iNOS up-regulation improves erectile function of diabetic rats. *J Urol* **2013**, *190*, 790-798, doi:10.1016/j.juro.2013.03.043.
28. Ma, L.; Jiang, K.; Jiang, P.; He, H.; Chen, K.; Shao, J.; Deng, G. Mechanism of Notch1-saRNA-1480 reversing androgen sensitivity in human metastatic castration-resistant prostate cancer. *Int J Mol Med* **2020**, *46*, 265-279, doi:10.3892/ijmm.2020.4597.
29. Huang, K.W.; Reebye, V.; Czysz, K.; Ciriello, S.; Dorman, S.; Reccia, I.; Lai, H.S.; Peng, L.; Kostomitsopoulos, N.; Nicholls, J., et al. Liver Activation of Hepatocellular Nuclear Factor-4α by Small Activating RNA Rescues Dyslipidemia and Improves Metabolic Profile. *Mol Ther Nucleic Acids* **2020**, *19*, 361-370, doi:10.1016/j.omtn.2019.10.044.
30. Zhu, Q.; Wu, X.; Huang, Y.; Tang, M.; Wu, L. Upregulation of FHIT gene expression in endometrial carcinoma by RNA activation. *Int J Clin Exp Pathol* **2020**, *13*, 1372-1380.
